# A Computational Model for Biomechanical Effects of Arterial Compliance Mismatch

**DOI:** 10.1155/2015/213236

**Published:** 2015-03-16

**Authors:** Fan He, Lu Hua, Li-jian Gao

**Affiliations:** ^1^Department of Mechanics, School of Science, Beijing University of Civil Engineering and Architecture, Beijing 100044, China; ^2^Key Laboratory of Clinical Trial Research in Cardiovascular Drugs, Ministry of Health, Fuwai Hospital, National Center for Cardiovascular Diseases, Chinese Academy of Medical Sciences, and Peking Union Medical College, Beijing 100037, China

## Abstract

*Background*. Compliance mismatch is a negative factor and it needs to be considered in arterial bypass grafting. *Objective*. A computational model was employed to investigate the effects of arterial compliance mismatch on blood flow, wall stress, and deformation. *Methods*. The unsteady blood flow was assumed to be laminar, Newtonian, viscous, and incompressible. The vessel wall was assumed to be linear elastic, isotropic, and incompressible. The fluid-wall interaction scheme was constructed using the finite element method. *Results*. The results show that there are identical wall shear stress waveforms, wall stress, and strain waveforms at different locations. The comparison of the results demonstrates that wall shear stresses and wall strains are higher while wall stresses are lower at the more compliant section. The differences promote the probability of intimal thickening at some locations. *Conclusions*. The model is effective and gives satisfactory results. It could be extended to all kinds of arteries with complicated geometrical and material factors.

## 1. Introduction

Arteriosclerosis is a frequent cardiovascular disease and has become one of the most leading causes of death in the modern society. Arteriosclerosis causes the deposition of low-density lipoprotein, atheroma, and cholesterol [1–3] and results in stenosis. It has been proven that the initiation and development of arteriosclerosis are related to hemodynamics and vessel wall mechanics [4–7]. Stenosis increases flow resistance in arteries and causes abnormal flow distributions, which may be related to thrombus formation and plaque rupture. Thus, stroke and heart attack are directly led [8]. It is well known that stenosis provokes abnormal flow and the resulting flow disorder further worsens the disease [9–14]. It is established that the arterial bypass grafting is effective in repairing the diseased arteries. Usually, the surgery is successful at the early stage of the operation; however there is a technical challenge for the surgeon due to the unacceptably high rate of postoperative occlusion [15]. Though the grafting has been performed extensively, the artificial or other self-body blood vessels are used in the grafting and thus a usual and troublesome problem is caused; that is, there is compliance mismatch in arterial bypass grafting. Compliance mismatch caused by different wall stiffness has been shown to reduce graft patency [16].

In this paper, a model composed of three arterial segments with different wall stiffness was constructed. Furthermore, a fluid-wall interaction was introduced to investigate blood flow and mechanical characteristics. In the model, the same walls at two ends were stiffer than the wall in the middle. This model was employed to simulate arterial compliance mismatch. The aim of this paper is to study the effects of arterial compliance mismatch on flow distributions, wall stresses, and deformations and find out the stiffness-induced differences of mechanical properties in the model. This study is helpful in making a detailed understanding of biomechanical effects of compliance mismatch. The understanding may be important in the selection of arterial grafts and in designing artificial blood vessels.

## 2. The Model

### 2.1. Geometry

**Figure 1:**
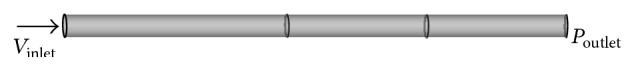
The geometry of the model.

Figure 1 shows the geometry of the model. The parameters of a human carotid artery were selected for the calculation [17]. The outer diameter was *D*_1_ = 10 mm and the inner diameter was *D*_0_ = 8 mm (a straight round vessel). The length of each wall was 50 mm. To minimize the influence of input velocity specification on the flow dynamics in the segments of interest, the segment in the upstream direction was extended [18]. An extension of 30 mm at the inflow side was added to the model where the inlet boundary condition was specified; thus, the total length was 180 mm. In this model, three arterial segments had the same diameter because we only wanted to investigate the effects of compliance mismatch. The effects of different geometrical structures were not considered here. The junction between two segments is related to geometrical structures and suture lines and angles. Therefore the mechanical properties of the junction were not considered in the paper and may be included in our future work.

### 2.2. Fluid and Wall Formulation

For the fluid model, the Navier-Stokes equations were used as the governing equations. Blood was modeled as an incompressible and Newtonian fluid, with a density of 1050 kg/m^3^ and a dynamic viscosity of 0.0035 Pa·s.

For the wall model, the linear elastic constitutive equations were used as the governing equations. The material constants used in the linear elastic relationship were Young's modulus and Poisson's ratio. Here, we made a compliance difference according to different values of Young's modulus. Young's modulus of the middle artery was 5 MPa and the moduli of the arteries at two ends were 200 MPa [19]. Poisson's ratio was 0.499.

**Figure 2:**
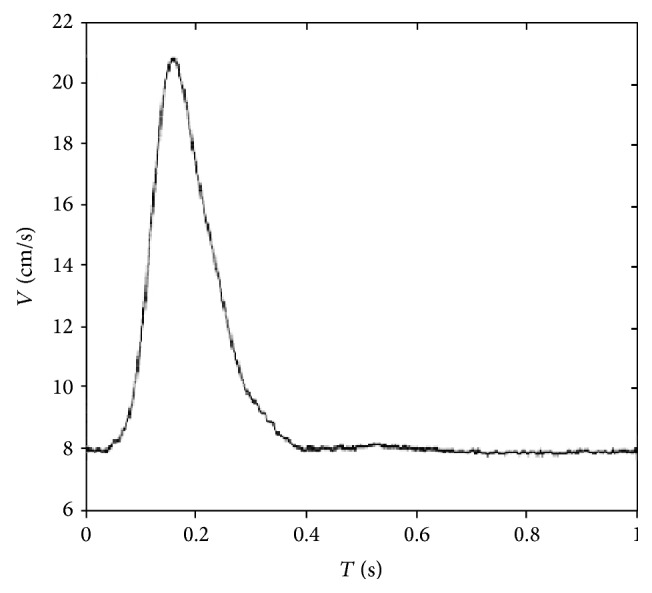
The map of velocity at the inlet.

Time-dependent flow condition measured by Gijsen et al. [20] (Figure 2) was applied at the inlet. The period of the flow waveform was *T* = 1 s. Then, the Reynolds number for the Newtonian fluid flow, defined as *Re* = *ρD*_0_ *V*/*μ*, varied from about 190 (diastole) to 500 (peak systole), while based on the period of the flow pulse, the Womersley number, defined as $\alpha =\left(D_{0}/2\right)\sqrt{2\pi \rho /T\mu }$, was equal to 5.5. At the outlet, a zero normal traction was applied. On the walls, we specify no-slip boundary conditions for the flow. The initial conditions in the whole field were assumed to be zero [21, 22]. In the structural mechanics part, as a boundary condition, we set that one end of the blood vessel was entirely fixed and the other end was free.

This fluid-structure interaction technique allows studying the arterial fluid mechanics by accounting for both the instantaneous fluid forces acting on the wall and the effects of the wall motion on the fluid dynamic field. Fluid forces, wall displacements, and velocities are transferred across the fluid-structure interface.

### 2.3. Numerical Methods

The fluid-wall interaction scheme was constructed using the finite element method [23]. The Arbitrary Lagrangian-Eulerian (ALE) formulation was used. It was an implicit procedure in time. A Crank-Nicolson time stepping scheme was employed. The fluid domain was discretized by unstructured tetrahedral four-node elements of 49461. The wall domain was discretized by hexahedral eight-node elements of 12960. Mesh convergence studies in which flow parameters and stress/displacement analyses with finer meshes were tested established that the results are independent of mesh density. Further increase of the mesh size did not significantly change the final results. We performed a maximum of 100 iterations within each time step of 0.01 s. Three cycles were required to achieve convergent and stable solutions for the transient analysis [24, 25]. The time step independency studies were performed (0.005 s and 0.008 s). The resulting values of the velocity from these time steps agreed to within 5%. The relative tolerance for all degrees of freedom was set to 0.0001.

## 3. Results and Discussion

### 3.1. Blood Flow Characteristics

**Figure 3:**
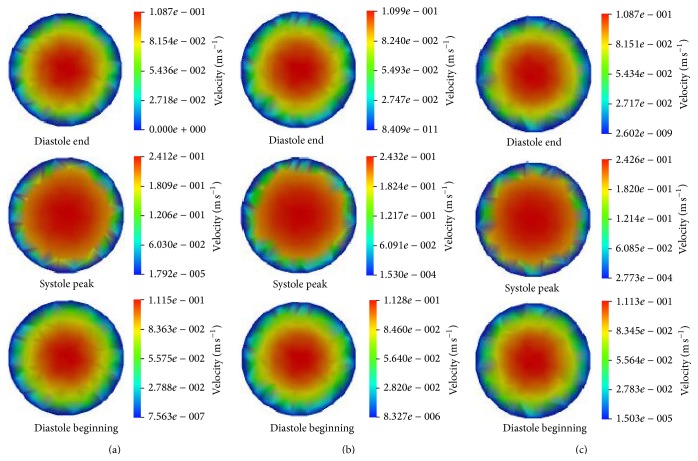
The velocity contours at different time. (a) *L* = 70 mm, (b) *L* = 100 mm, and (c) *L* = 140 mm.

Figure 3 presents the calculated velocity contours at three time points, that is, diastole end, systole peak, and diastole begin. In Figure 3, *L* represents the distance away from the inlet. Thus, *L* = 70 mm, 100 mm, and 140 mm denote the locations of the beginning, middle, and ending tubes in Figure 1, respectively. The entrance effects are considered when selecting the locations; therefore it is ensured that *L* = 70 mm is excluded in the entrance zone.

It can be seen that the velocity distributions at different locations are almost identical at the same time. The maximum velocity values at *L* = 100 mm are higher than those at other locations. The velocity profiles in Figure 3 are not axisymmetric. The reason is that the deformation of the tube is accompanied with buckling. The buckling breaks the symmetry. The reason of the buckling could be found in Han's work [26].

**Figure 4:**
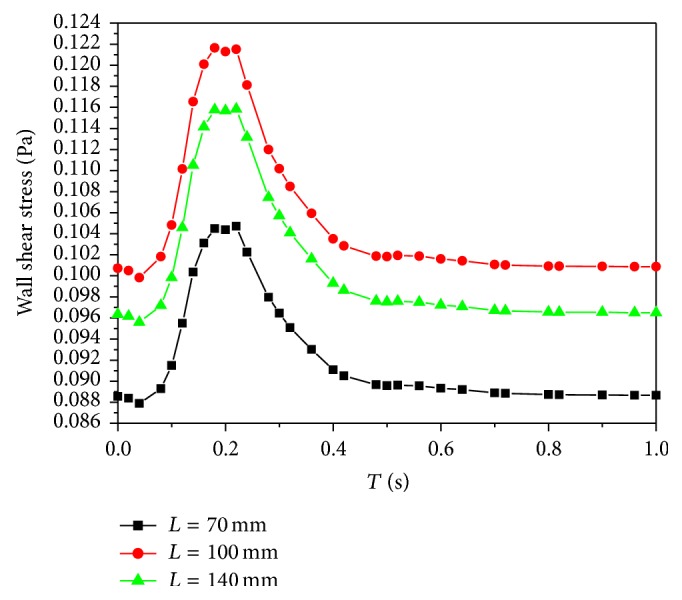
The distributions of time-dependent wall shear stresses.

**Figure 5:**
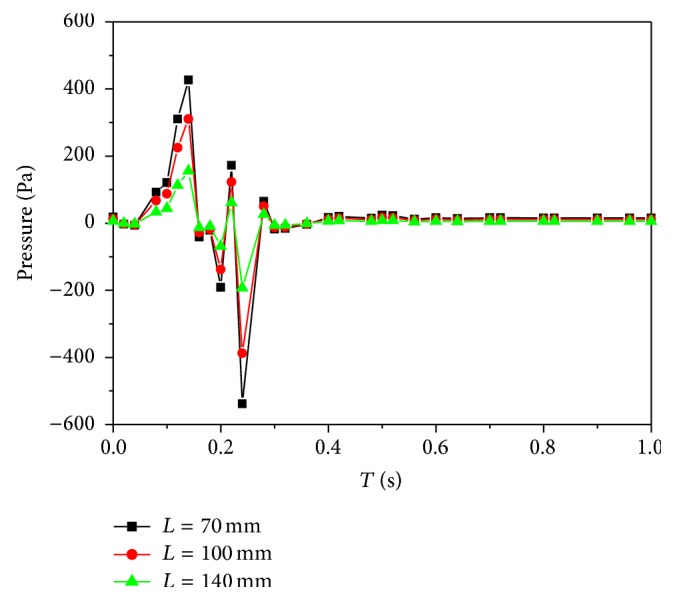
The distributions of time-dependent pressures.

Here, the maximum wall shear stresses at three locations are presented (Figure 4). The distributions of wall shear stresses closely resemble the inlet velocity waveform. However obviously, the wall shear stresses at *L* = 100 mm are highest in Figure 4. The characteristics of time-dependent pressures of the three locations are very much similar as shown in Figure 5. According to the results, there is the evidence that a variation of Young's modulus (arterial compliance) does not change the flow distributions. These are in agreement with the results obtained by Valencia and Solis [19].

As we know, the increasing residence time of atherogenic particles, such as platelets, leukocytes, and macrophages, could increase the probability of deposition or adhesion of blood particles with the arterial wall. The residence time is correlated with wall shear stress. Low wall shear stress is prone to increase the residence time and it can promote plaques formation and intimal thickening [27]. Accordingly, the wall shear stresses at the middle wall are more advantageous than those at the beginning and ending walls; that is, stiffer wall may induce lower wall shear stresses and promote arterial diseases more easily. Therefore the arterial compliance mismatch may bring disadvantageous effects on wall shear stresses and result in the occurrence of the particle deposition at stiffer arterial segments.

### 3.2. Wall Mechanics

**Figure 6:**
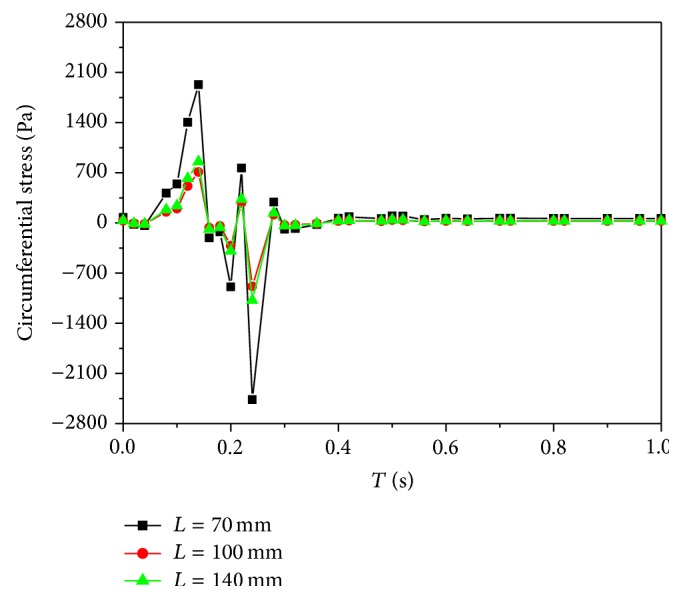
The distributions of time-dependent circumferential stresses.

**Figure 7:**
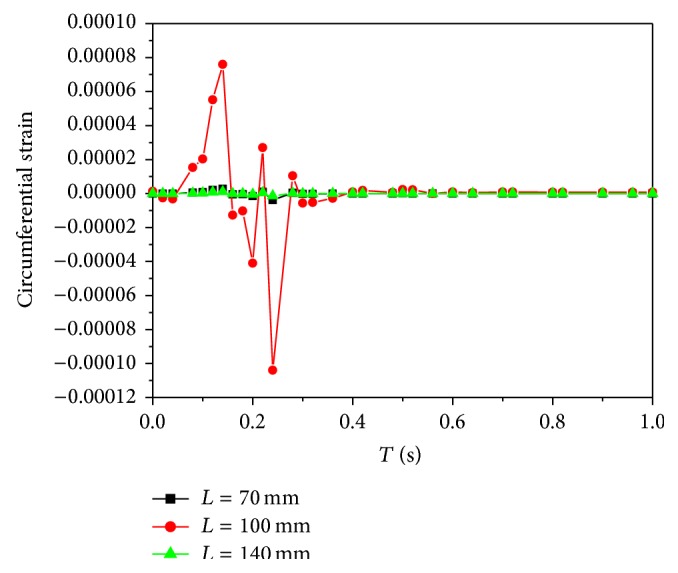
The distributions of time-dependent circumferential strains.

The stresses and strains in an arterial wall are caused by both blood pressure and wall shear. It is known that circumferential stresses and strains are directly related to the remodeling of arterial walls and affect the structure and physiological function of arterial walls. Therefore, the circumferential stresses and strains of the inner walls at three locations are concerned in this study. The time dependence of the circumferential stresses is the same as that of the pressures (Figure 6). Meanwhile, the relationship between the stress and strain is linear elastic. Thus, the distributions of the circumferential strains are similar to those of the circumferential stresses (Figure 7).

The absolute maximum and minimum values of the circumferential stresses at *L* = 100 mm are lowest as shown in Table 1. It is known that a high stress level tends to induce a concentrated stress. The structures of cells and tissues enduring long-term high stresses would be changed. Therefore a high stress level is harmful. From the point, low circumferential stresses adapt to keep arterial tissues growing normally and healthily.

The absolute maximum and minimum values of the circumferential strains at *L* = 100 mm are highest as shown in Table 2. Moreover, the strains at *L* = 100 mm are much higher compared to other locations, so that the strain distributions at *L* = 70 mm and 140 mm are unclear in Figure 7.

Cyclic strain may play a role in atherosclerosis. It is possible that there exists a correlation between high cyclic strain levels and enhanced macromolecular permeability, in turn leading to atherosclerotic inflammation [28]. Straining with a large strain magnitude results in a negative effect on the mechanical properties of the tissue [29]. Though the strain magnitude is very small in Table 2, the strain distributions are nonuniform. The strain magnitude at *L* = 100 mm is higher than that at other locations. If the outlet pressure is enhanced, the strain magnitude is also increased as a result. In that way, the strain magnitude at *L* = 100 mm could affect the mechanical properties of the tissue unfavorably when it attains to a degree. The strains at other locations are more advantageous. From this aspect, the strains in the middle artery are not preponderant in the tissue growth.

### 3.3. Limitations of the Model

Several simplifying assumptions were made in the current analysis, which ignored anisotropy, non-linear elastic properties of the arterial wall, non-Newtonian blood, and so forth. All these higher order effects can be considered in future studies.

## 4. Conclusions

A computational model for biomechanical effects of arterial compliance mismatch on blood flow and mechanical characteristics has been constructed. The flow distributions and wall stresses and deformations based on the model have been performed numerically. The results indicate that the arterial compliance mismatch could induce the differences of mechanical characteristics between different locations. The differences promote the probability of intimal thickening at some locations. Compliance mismatch is a negative factor and it needs to be considered cautiously in arterial bypass grafting. Consequently, the avoidance of compliance mismatch could enhance graft patency. The constructed model in this paper gives satisfactory results and may be better in expressing biomechanical properties of arterial compliance mismatch. The computational model is effective and could be extended to all kinds of arteries with complicated geometrical and material factors.

## Figures and Tables

**Table 1:** The maximum and minimum circumferential stresses at three locations.

*L* (mm)	Maximum stress (Pa)	Minimum stress (Pa)
70	2088.38	−2465.67
100	760.663	−886.142
140	919.78	−1078.18

**Table 2:** The maximum and minimum circumferential strains at three locations.

*L* (mm)	Maximum strain	Minimum strain
70	3.10779*e* − 6	−3.83545*e* − 6
100	8.49064*e* − 5	−1.04459*e* − 4
140	1.13953*e* − 6	−1.39365*e* − 6
